# A High Phosphorus Diet Affects Lipid Metabolism in Rat Liver: A DNA Microarray Analysis

**DOI:** 10.1371/journal.pone.0155386

**Published:** 2016-05-17

**Authors:** Sunwoo Chun, Takeshi Bamba, Tatsuya Suyama, Tomoko Ishijima, Eiichiro Fukusaki, Keiko Abe, Yuji Nakai

**Affiliations:** 1 Graduate School of Agricultural and Life Sciences, The University of Tokyo, Tokyo, Japan; 2 Department of Biotechnology, Graduate School of Engineering, Osaka University, Suita, Osaka, Japan; 3 Project on Health and Anti-aging, Kanagawa Academy of Science and Technology Life Science & Environment Research Center, Kawasaki, Kanagawa, Japan; University of Basque Country, SPAIN

## Abstract

A high phosphorus (HP) diet causes disorders of renal function, bone metabolism, and vascular function. We previously demonstrated that DNA microarray analysis is an appropriate method to comprehensively evaluate the effects of a HP diet on kidney dysfunction such as calcification, fibrillization, and inflammation. We reported that type IIb sodium-dependent phosphate transporter is significantly up-regulated in this context. In the present study, we performed DNA microarray analysis to investigate the effects of a HP diet on the liver, which plays a pivotal role in energy metabolism. DNA microarray analysis was performed with total RNA isolated from the livers of rats fed a control diet (containing 0.3% phosphorus) or a HP diet (containing 1.2% phosphorus). Gene Ontology analysis of differentially expressed genes (DEGs) revealed that the HP diet induced down-regulation of genes involved in hepatic amino acid catabolism and lipogenesis, while genes related to fatty acid β-oxidation process were up-regulated. Although genes related to fatty acid biosynthesis were down-regulated in HP diet-fed rats, genes important for the elongation and desaturation reactions of omega-3 and -6 fatty acids were up-regulated. Concentrations of hepatic arachidonic acid and eicosapentaenoic acid were increased in HP diet-fed rats. These essential fatty acids activate peroxisome proliferator-activated receptor alpha (PPARα), a transcription factor for fatty acid β-oxidation. Evaluation of the upstream regulators of DEGs using Ingenuity Pathway Analysis indicated that PPARα was activated in the livers of HP diet-fed rats. Furthermore, the serum concentration of fibroblast growth factor 21, a hormone secreted from the liver that promotes fatty acid utilization in adipose tissue as a PPARα target gene, was higher (*p* = 0.054) in HP diet-fed rats than in control diet-fed rats. These data suggest that a HP diet enhances energy expenditure through the utilization of free fatty acids released via lipolysis of white adipose tissue.

## Introduction

Phosphorus is a vital mineral in many biological processes and exists in the form of inorganic phosphates in the body. Phosphorus is abundant in many foods as natural phosphorus and phosphate-containing food additives. In many countries, dietary phosphorus intake is about 2–3-fold higher [1–3] than the recommended level (700 mg/d) [4]. Intake of phosphorus from phosphate additives has gradually increased over the past several decades owing to lifestyle changes [5–7]. Administration of a high phosphorus (HP) diet causes disorders of renal function, bone metabolism, and vascular cell function [8–10]. Furthermore, recent reports suggest that HP diets accelerate aging [11, 12].

Phosphate homeostasis is primarily regulated by three organs: the small intestine functions in phosphate absorption, the kidney functions in phosphate excretion, and bone acts as a phosphate reservoir. These organs are coordinately regulated in response to changes in dietary and serum phosphate levels. Thus, previous studies of the influence of a HP diet, especially with regards to phosphate homeostasis control, have focused on these pivotal organs. We previously demonstrated that rats fed on a HP diet induces expression of genes related to calcification, fibrillization, and inflammation in the kidney, and we also obtained new information on phosphate homeostasis regulation by performing DNA microarray analysis [13]. However, little is known about the effects of a HP diet on other organs that are considered to be irrelevant to the regulation of phosphate homeostasis.

The liver is a key organ that plays important roles in energy metabolism such as carbohydrate, lipid, and amino acid metabolism. Dietary phosphates absorbed by the small intestine are transported to the liver via the portal vein. Specific sodium-phosphate co-transporters, which are regulated in response to changes in dietary and serum phosphate levels, are reportedly present in hepatocytes [14]. Therefore, a HP diet likely influences the liver, as reported previously in papers showing that a HP diet decreases protein synthesis in rat liver [15] and enhances liver development in young mice [16]. These findings strongly indicate that HP diets affect liver function, although the conditions used for animal experiments differed between these previous studies.

Thus, we investigated the global effects of a HP diet on the liver, especially with regards to energy metabolism. To investigate overall metabolic alterations, gene expression in the livers of HP diet-fed rats was assessed by DNA microarray analysis. Here we report that a HP diet affects the expression of genes involved in lipid and amino acid metabolism.

## Results

### Food intake, body weight, and phosphorus balance

The effects of the HP diet on food intake, body weight, serum phosphorus concentration, and phosphorus balance were identical to those previously reported [13], because the animals used in the present study were the same as those used in the previous study. Briefly, there were no significant differences in food intake, body weight, or phosphorous retention between control and HP diet-fed rats, except that HP diet-fed animals ingested and excreted a higher amount of phosphorus. HP diet-fed rats showed significantly lower serum phosphorus concentrations, while there were no differences in serum calcium concentrations between the two groups of rats.

### DNA microarray analysis of gene expression

Microarray data quantified by Factor Analysis for Robust Microarray Summarization (FARMS) [17] were subjected to principal component analysis. Rats fed the HP diet and the control diet formed clusters that were distinct from each other (S1 Fig). This result suggests that the HP diet significantly influences the gene expression profile in rat liver.

To identify differentially expressed genes (DEGs) in response to the HP diet, the rank products (RP) method [18] was applied to FARMS-quantified data. We identified 353 up-regulated probe sets (271 unique genes) and 496 down-regulated probe sets (372 unique genes) in the HP diet group compared to the control diet group, with a false discovery rate (FDR) of <0.05. The full list of up- and down-regulated probe sets is shown in S1 Table.

### Gene Ontology analysis

**Fig 1:**
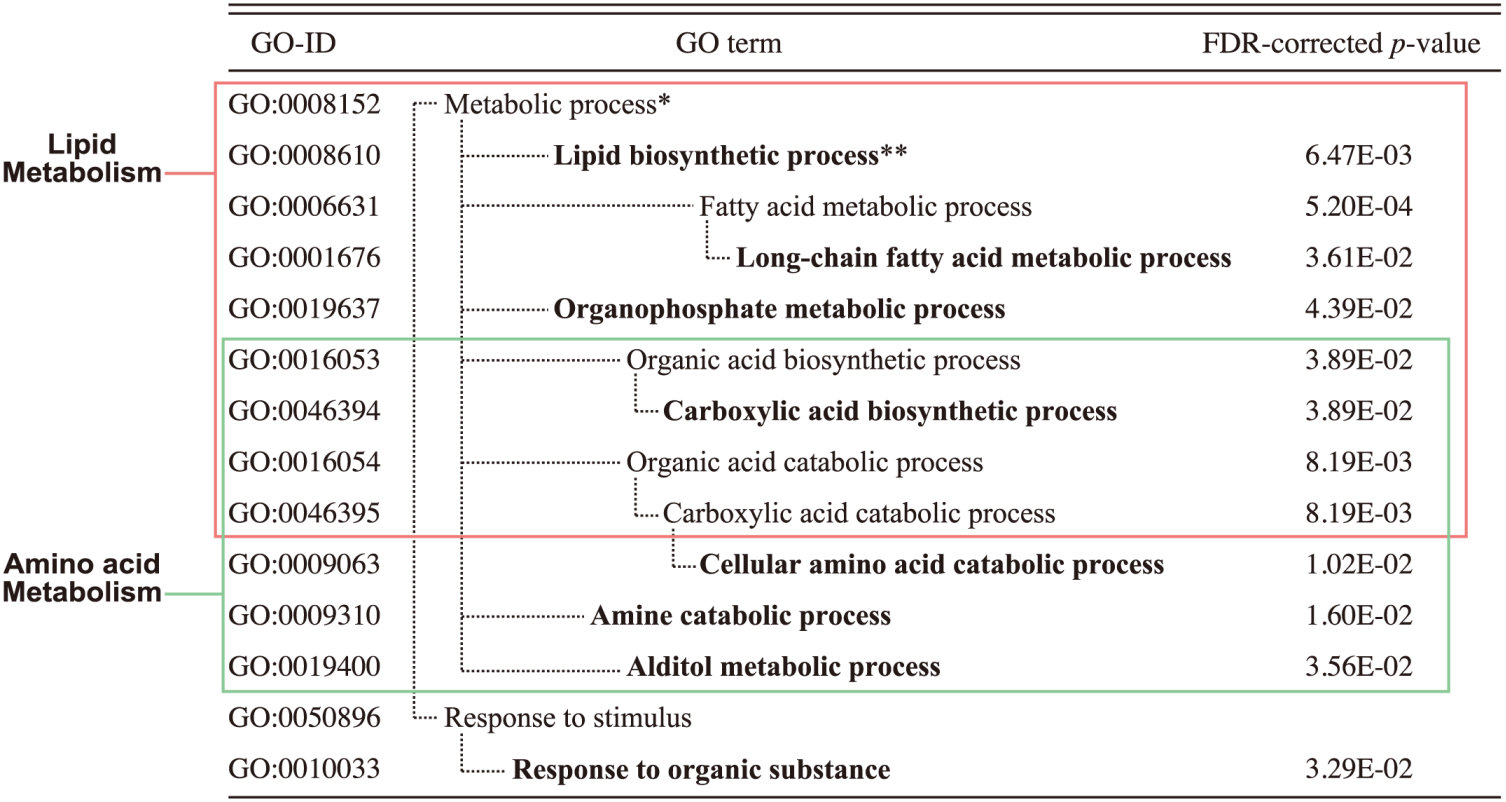
Gene Ontology (GO) terms that were significantly enriched (*p* < 0.05) in 643 differentially expressed genes (DEGs) in response to a high phosphorous (HP) diet. * GO terms with no assigned *p*-values indicate that the values are not significant. ** GO terms in the deepest level of hierarchy are shown in bold.

DEGs were classified into functional categories according to Gene Ontology (GO) analysis, and GO terms enriched among the DEGs are summarized in Fig 1. The over-represented GO terms were classified into “metabolic process” and “response to stimulus” groups, where those in the former group were further classified into two mutually overlapping major GO term clusters: one related to amino acid metabolism and the other to lipid metabolism (Fig 1).

#### Amino acid metabolism-related GO terms

Genes associated with GO terms related to amino acid metabolism were divided into two groups based on their functions: amino acid catabolism and involvement in the urea cycle (S2 Table).

**Fig 2:**
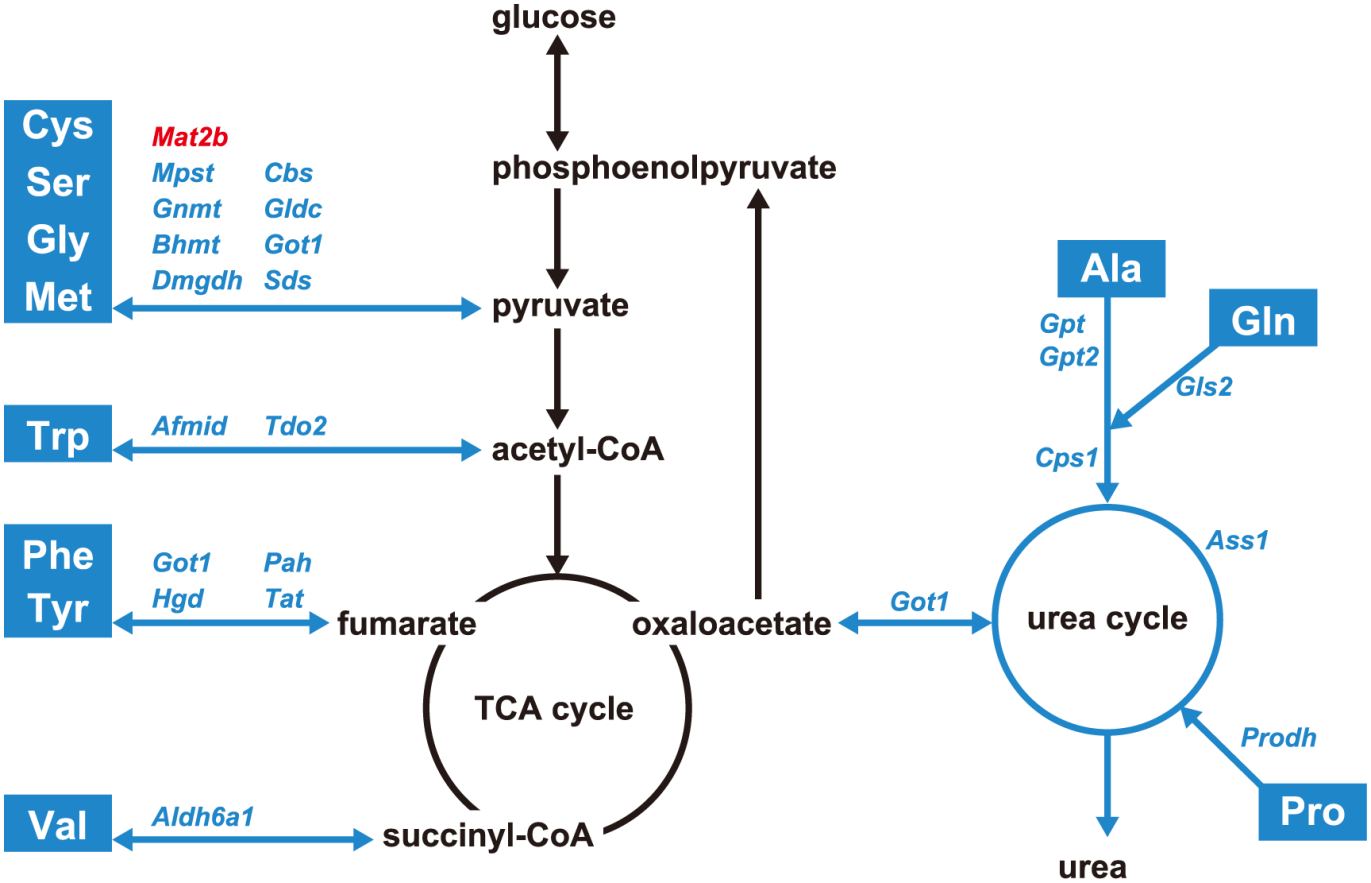
Altered expression of genes involved in the amino acid metabolic pathway in response to the high phosphorous (HP) diet. The HP diet induced decreased hepatic expression of enzymes involved in amino acid catabolism. Enzymes and factors whose mRNA expression is higher and lower in the livers of HP diet-fed rats are shown in red and blue, respectively, in comparison to their expression in the livers of control diet-fed rats. Also see S2 Table for DEGs related to amino acid metabolism. Amino acids are represented by white letters and down-regulated pathways are represented by blue arrows. TCA, tricarboxylic acid.

Mapping of the DEGs related to amino acid metabolic pathways revealed that their hepatic expression was lower in rats fed the HP diet than in rats fed the control diet, with the exception of *Mat2b* (Fig 2). HP diet-fed rats exhibited lower hepatic expression of many enzymes related to catabolism of amino acids (Cys, Ser, Gly, Met, Trp, Phe, Tyr, and Val), and the amino acid catabolites are used to generate energy via gluconeogenesis or ketogenesis. These genes were *Got1*, *Mpst*, *Sds*, *Cbs*, *Bhmt*, *Gldc*, *Gnmt*, *Dmgdh*, *Tdo2*, *Afmid*, *Pah*, *Tat*, *Hgd*, and *Aldh6a1*. Furthermore, genes related to the urea cycle were down-regulated in the livers of rats fed the HP diet. These genes included *Cps1*, which catalyzes the committed step of the urea cycle, and *Got1*, *Gpt*, *Gpt2*, *Prodh*, and *Gls2*, which regulate the entry of amino acid catabolites into this cycle.

#### Lipid metabolism-related GO terms

**Fig 3:**
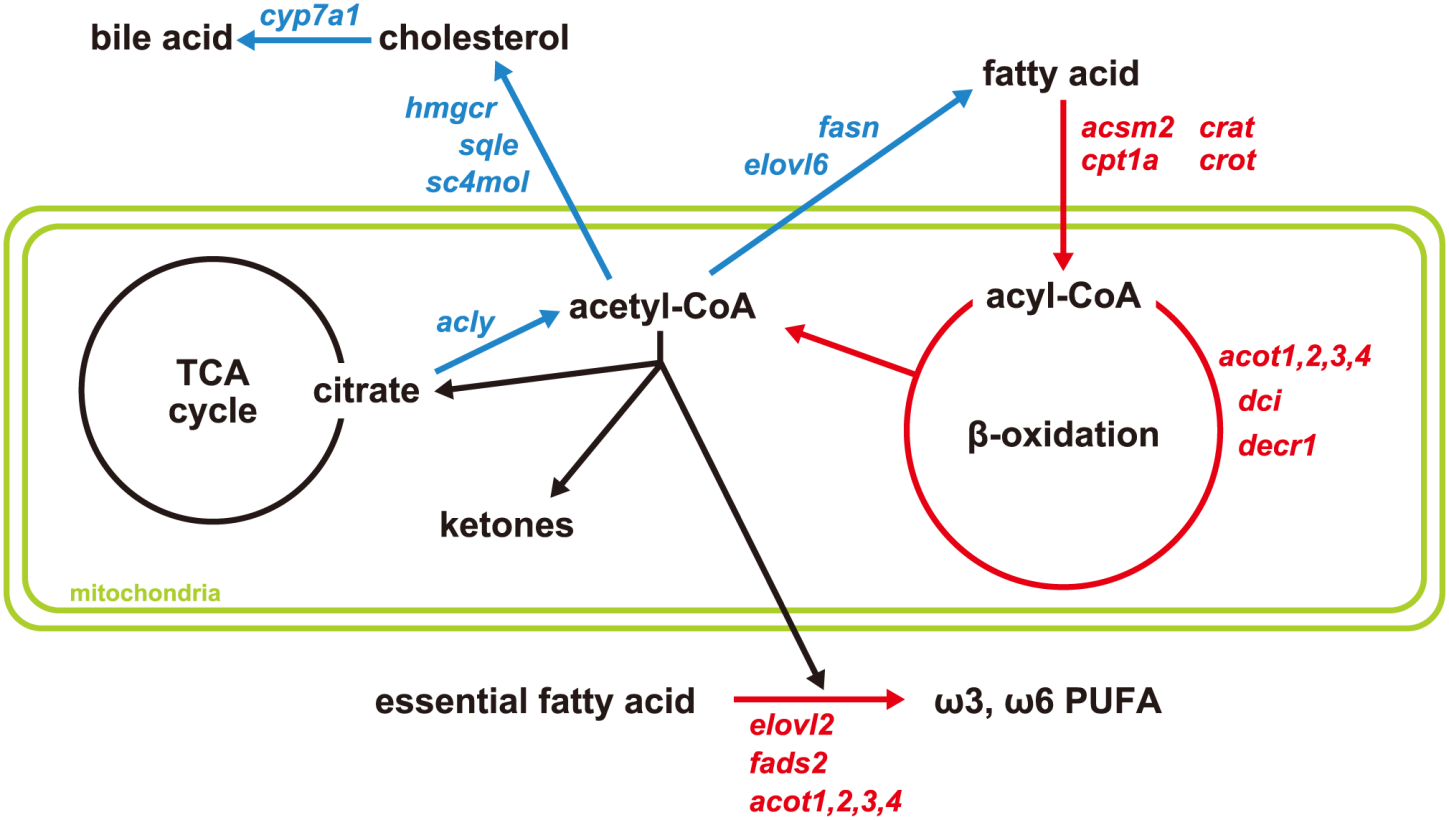
Altered expression of genes involved in the lipid metabolic pathway in response to the high phosphorous (HP) diet. The HP diet induced alterations in the hepatic expression of enzymes involved in lipid catabolism and biosynthesis. Enzymes and factors whose mRNA expression is higher and lower in the livers of HP diet-fed rats are shown in red and blue, respectively, in comparison to their expression in the livers of control diet-fed rats. See Table 1 for DEGs related to lipid metabolism. Up- and down-regulated pathways are represented with red and blue arrows, respectively. PUFA, polyunsaturated fatty acid; TCA, tricarboxylic acid.

Genes associated with GO terms related to lipid metabolism were categorized into four groups: fatty acid β-oxidation, fatty acid biosynthesis, cholesterol metabolism, and phospholipid metabolism (Tables 1 and 2; Fig 3).

**Table 1:** Genes related to fatty acid β-oxidation or fatty acid biosynthesis whose expression was altered in response to the HP diet.

GO term	Gene Symbol	Gene Title	Average Difference	Expression
**Fatty acid β-oxidation**	*Cpt1a*	carnitine palmitoyltransferase 1a, liver	3.93E-01	up
	*Crot*	carnitine O-octanoyltransferase	3.66E-01	up
	*Crat*	carnitine acetyltransferase	5.65E-01	up
	*Acsm2*	acyl-CoA synthetase medium-chain family member 2	3.91E-01	up
	*Acot1*	acyl-CoA thioesterase 1	1.66E+00	up
	*Acot2*	acyl-CoA thioesterase 2	5.95E-01	up
	*Acot3*	acyl-CoA thioesterase 3	4.10E-01	up
	*Acot4*	acyl-CoA thioesterase 4	8.06E-01	up
	*Abhd5*	abhydrolase domain containing 5	2.34E-01	up
	*Hadhb*	hydroxyacyl-Coenzyme A dehydrogenase, beta subunit	1.54E-01	up
	*Dci*	dodecenoyl-Coenzyme A delta isomerase	4.37E-01	up
	*Ech1*	enoyl coenzyme A hydratase 1, peroxisomal	5.33E-01	up
	*Decr1*	2,4-dienoyl CoA reductase 1, mitochondrial	2.67E-01	up
	*Acacb*	acetyl-Coenzyme A carboxylase beta	2.48E-01	up
	*Cyp4a1 / Cyp4a10*	cytochrome P450, family 4, subfamily a, polypeptide 1; polypeptide 10	4.28E-01	up
	*Cyp4a2 / Cyp4a3*	cytochrome P450, family 4, subfamily a, polypeptide 2; polypeptide 3	3.13E-01	up
	Fabp1*	fatty acid binding protein 1, liver	1.04E-01	up
	*Fabp2*	fatty acid binding protein 2, intestinal	2.73E-01	up
	Fabp7*	fatty acid binding protein 7, brain	4.39E-01	up
	*Hacl1*	2-hydroxyacyl-CoA lyase 1	5.37E-01	up
**Fatty acid biosynthesis**	*Acacb*	acetyl-Coenzyme A carboxylase beta	2.48E-01	up
	*Fads2*	fatty acid desaturase 2	3.32E-01	up
	*Prkab1*	protein kinase, AMP-activated, beta 1 non-catalytic subunit	2.18E-01	up
	Elovl2*	elongation of very long chain fatty acids (FEN1/Elo2, SUR4/Elo3, yeast)-like 2	3.05E-01	up
	*Acly*	ATP citrate lyase	-2.40E-01	down
	*Fasn*	fatty acid synthase	-2.61E-01	down
	*Elovl6*	ELOVL family member 6, elongation of long chain fattyacids (yeast)	-5.30E-01	down
	*Srebf1*	sterol regulatory element binding transcription factor 1	-3.59E-01	down

*These DEGs are not included in the GO terms but were considered to be related to the GO term based on the KEGG pathway or other GO annotations.

**Table 2:** Genes related to cholesterol or phospholipid metabolism whose expression was altered in response to the HP diet.

GO term	Gene Symbol	Gene Title	Average Difference	Expression
**Cholesterol metabolic process**	*Hmgcs1*	3-hydroxy-3-methylglutaryl-Coenzyme A synthase 1 (soluble)	3.67E-01	up
	Fxr*	nuclear receptor subfamily 1, group H, member 4	1.89E-01	up
	*Prkab1*	protein kinase, AMP-activated, beta 1 non-catalytic subunit	2.18E-01	up
	Hnf4a*	hepatocyte nuclear factor 4, alpha	4.54E-01	up
	*Hmgcr*	3-hydroxy-3-methylglutaryl-Coenzyme A reductase	-4.01E-01	down
	Sqle*	squalene epoxidase	-2.08E-01	down
	*Sc4mol*	sterol-C4-methyl oxidase-like	-2.10E-01	down
	*Cyp7a1*	cytochrome P450, family 7, subfamily a, polypeptide 1	-1.22E+00	down
	Shp (Nr0b2)*	nuclear receptor subfamily 0, group B, member 2	-2.82E-01	down
	Lrh1 (NR5A2)*	similar to FTZ-F1 beta1 protein; nuclear receptor subfamily 5, group A, member 2	-2.18E-01	down
	*Abca1*	ATP-binding cassette, sub-family A (ABC1), member 1	-2.09E-01	down
	*Pcsk9*	proprotein convertase subtilisin/kexin type 9	-1.69E-01	down
	*Srebf1*	sterol regulatory element binding transcription factor 1	-3.59E-01	down
	*Insig1*	similar to Insulin-induced gene 1 protein (INSIG-1)	-5.02E-01	down
	*Angptl3*	angiopoietin-like 3	-1.66E-01	down
	Atp8b1*	ATPase, Class I, type 8B, member 1	-2.42E-01	down
**Phospholipid metabolic process**	*Agpat9*	1-acylglycerol-3-phosphate O-acyltransferase 9	7.61E-01	up
	*Gpd1*	glycerol-3-phosphate dehydrogenase 1 (soluble)	1.49E-01	up
	*Gpd2*	glycerol-3-phosphate dehydrogenase 2, mitochondrial	2.07E-01	up
	*Pla2g15*	phospholipase A2, group XV	2.93E-01	up
	*Cds1*	CDP-diacylglycerol synthase 1	2.55E-01	up
	*Chka*	choline kinase alpha	2.40E-01	up
	*Lpcat3*	lysophosphatidylcholine acyltransferase 3	2.99E-01	up
	*Pigx*	phosphatidylinositol glycan anchor biosynthesis, class X	2.60E-01	up
	*Gpld1*	glycosylphosphatidylinositol specific phospholipase D1	-2.53E-01	down
	*Pik3r1*	phosphoinositide-3-kinase, regulatory subunit 1 (alpha)	-2.91E-01	down
	*Etnk1*	ethanolamine kinase 1	-2.59E-01	down
	*Gpam*	glycerol-3-phosphate acyltransferase, mitochondrial	-1.94E-01	down
	*Pi4k2a*	phosphatidylinositol 4-kinase type 2 alpha	-2.64E-01	down
	*Pigu*	phosphatidylinositol glycan anchor biosynthesis, class U	-2.32E-01	down

*These DEGs are not included in the GO terms but were considered to be related to the GO term based on the KEGG pathway or other GO annotations.

Among the fatty acid β-oxidation group, DEGs involved in the oxidation of fatty acids into acetyl-CoA were up-regulated in the livers of HP diet-fed rats. Expression of *Cpt1a*, which encodes a rate-limiting enzyme for β-oxidation, was up-regulated, concomitant with the up-regulation of many genes involved in fatty acid degradation (*Crat*, *Crot*, *Acot1*, *Acot2*, *Acot3*, *Acot4*, *Decr1*, *Dci*, and *Acsm2*) (Fig 3). Aside from the genes presented in Fig 3, several genes involved in the control of β-oxidation, including *Acacb*, *Abhd5*, *Hacl1*, *Ech1*, *Cyp4a1*/*Cyp4a10*, *Cyp4a2*/*Cyp4a3*, *Fabp2*, and *Fabp7*, were also up-regulated (Table 1). Moreover, *Fgf21*, which encodes a key factor fibroblast growth factor 21 (FGF21) for energy metabolism, was significantly up-regulated in the livers of rats fed the HP diet.

On the other hand, several genes encoding enzymes or transcription factors involved in fatty acid biosynthesis, including *Fasn*, *Elovl6*, *Acly*, and *Srebf1*, were down-regulated in the livers of HP diet-fed rats (Table 1; Fig 3). By contrast, genes encoding elongase and desaturase enzymes (*Elovl2* and *Fads2*) for essential fatty acids were up-regulated in the livers of rats fed the HP diet.

Many genes encoding enzymes important for cholesterol metabolism were up- or down-regulated (Table 2; Fig 3). Genes encoding Hmgcs1, the enzyme that produces HMG-CoA from acetyl-CoA, and Fxr, the nuclear receptor that binds bile acids (catabolites of cholesterol), were up-regulated in the livers of rats fed the HP diet (Table 2). On the other hand, many genes encoding enzymes important for cholesterol biosynthesis were down-regulated in the livers of HP diet-fed rats. *Sqle* and *Sc4mol*, which regulate cholesterol synthesis, were down-regulated following a decrease in the expression of *Hmgcr*, which encodes a rate-limiting enzyme. Specific genes related to cholesterol metabolism were also down-regulated in the livers of HP diet-fed rats, such as *Cyp7a1*, which encodes an enzyme that regulates the rate-limiting step of cholesterol catabolism, *Shp* and *Lrh1*, which encode transcription factors located downstream of Fxr, and *Atp8b1*, which encodes a transporter of bile acids.

The fourth DEG cluster associated with lipid metabolism included many genes related to phospholipid metabolism (Table 2). Genes encoding enzymes that catalyze binding of phospholipids and acyl-CoA, including *Agpat9* and *Lpcat3*, were up-regulated in the livers of rats fed the HP diet, whereas *Gpam* was down-regulated. Genes encoding enzymes involved in phosphatidylcholine synthesis, including *Chka* and *Pla2g15*, were up-regulated in the livers of HP diet-fed rats. *Cds1*, which encodes an enzyme that catalyzes phosphatidylinositol synthesis, was also up-regulated in the livers of rats fed the HP diet, whereas *Pik3r1* and *Pi4k2a* were down-regulated.

On the other hand, some genes related to phospholipid metabolism were down-regulated in the livers of rats fed the HP diet. These included *Etnk1*, which encodes an enzyme involved in phosphatidylethanolamine synthesis, *Pigx*, *Pigu*, and *Gpld1*, which encode enzymes involved in glycosylphosphatidylinositol anchor metabolism, and *Gpd1* and *Gpd2*, which encode enzymes involved in lipid and carbohydrate metabolism.

### Biochemical analysis of lipids in serum and liver

To confirm the results from DNA microarray analysis regarding changes in lipid metabolism in the livers of rats fed the HP diet, we measured lipid biochemical parameters in the serum and liver as well as the proportions of fatty acids in the liver.

There were no significant differences between rats fed the HP diet and those fed the control diet in terms of the serum concentrations of lipids, total cholesterol (T-CHO), esterified cholesterol (E-CHO), triacylglycerol (TG), phospholipid (PL), low-density lipoprotein cholesterol (LDL-C), high-density lipoprotein cholesterol (HDL-C), non-esterified fatty acid (NEFA), and total ketone body (T-KB) (S2 Fig). There were also no significant differences in serum glycerol concentration (S3 Fig).

**Fig 4:**
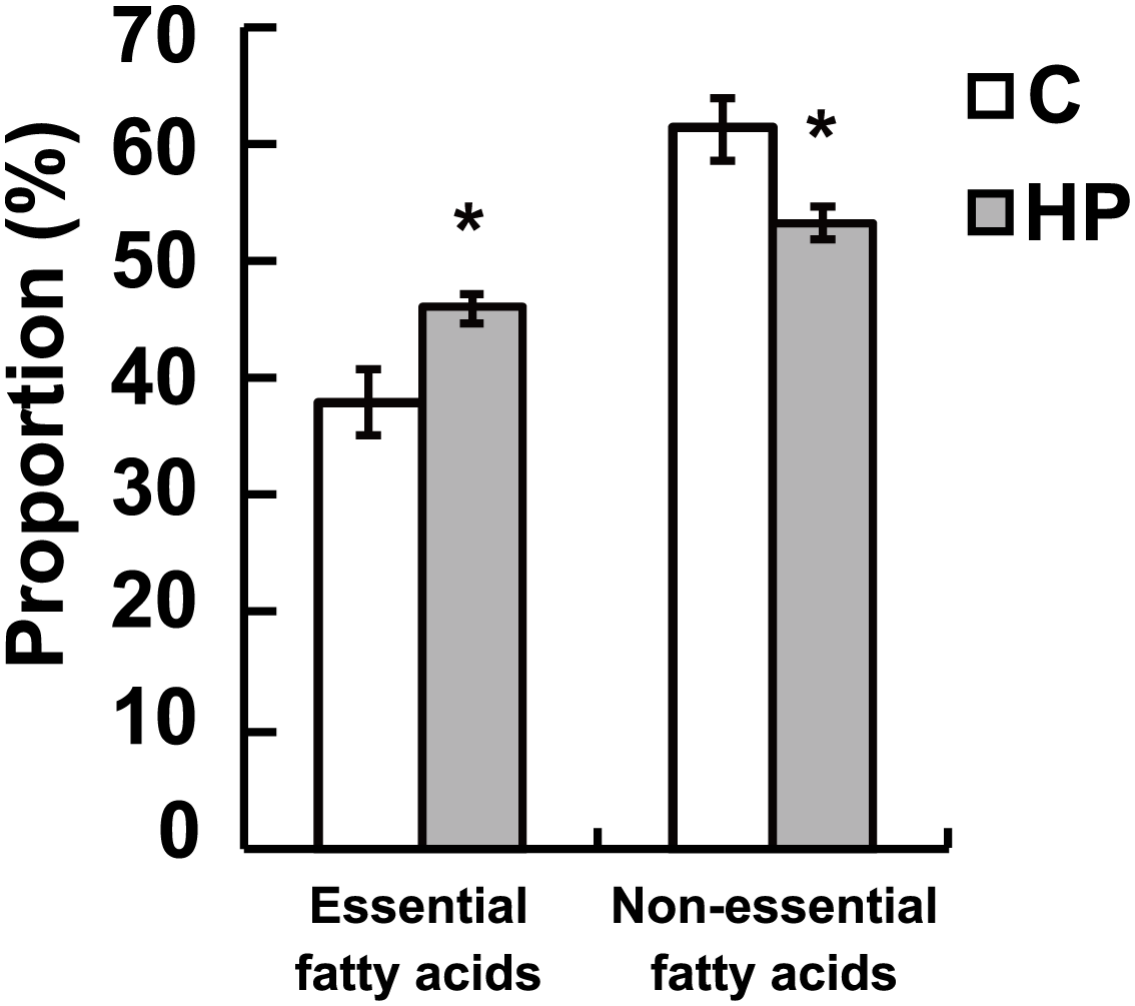
Altered hepatic fatty acid composition in response to the high phosphorous (HP) diet. Proportions of essential and non-essential fatty acids in the liver. The percent compositions were calculated based on the fatty acid content from 32 detected peaks. Data represent means ± standard error (*n* = 6). * *p* < 0.05 vs. control group. C, rats fed the control diet; HP, rats fed the HP diet.

There were no significant differences between rats fed the HP diet and those fed the control diet in terms of hepatic concentrations of PL, T-CHO, E-CHO, and TG (data not shown). However, the proportion of omega-3 and -6 essential fatty acids in the liver was significantly higher in HP diet-fed rats than in control diet-fed rats (Fig 4). Among non-essential fatty acids, the percentages of C8:0, C12:0, C14:0, C14:1, C16:0 (palmitic acid), C16:1 (palmitoleic acid), and C18:1 (n-9) (oleic acid) were lower, whereas the percentages of C17:0, C18:0 (stearic acid), C20:0, C22:0, and C23:0 were significantly higher, in the livers of HP diet-fed rats than in those of control diet-fed rats (Table 3). Among essential fatty acids, the proportions of C18:3 (n-6) (γ-linolenic acid), C20:4 (n-6) (arachidonic acid), and C20:5 (n-3) (eicosapentaenoic acid; EPA) in the liver were significantly higher in HP diet-fed rats than in control diet-fed rats (Table 4).

**Table 3:** Alterations in hepatic non-essential fatty acid composition in response to the HP diet.

	composition(%)	
Fatty acid	C	HP
Caprylic acid (C8:0)	0.0045 ± 0.0005	0.0026 ± 0.0006*
Capric acid (C10:0)	0.0074 ± 0.0005	0.0077 ± 0.0005
Undecanoic acid (C11:0)	0.0028 ± 0.0003	0.0035 ± 0.0004
Lauric acid (C12:0)	0.0191 ± 0.0007	0.0158 ± 0.0007*
Myristic acid (C14:0)	0.7227 ± 0.0754	0.4672 ± 0.0441*
Myristoleic acid (C14:1)	0.0393 ± 0.0086	0.0171 ± 0.0047*
Pentadecanoic acid (C15:0)	0.1087 ± 0.0057	0.1101 ± 0.0040
*cis*-10-Pentadecenoic acid (C15:1)	0.0044 ± 0.0011	0.0038 ± 0.0007
Palmitic acid (C16:0)	26.88 ± 1.60	22.46 ± 0.54*
Palmitoleic acid (C16:1)	5.414 ± 0.772	2.709 ± 0.317*
Heptadecanoic acid (C17:0)	0.1493 ± 0.0089	0.2075 ± 0.0118*
*cis*-10-Heptadecenoic acid (C17:1)	0.1076 ± 0.0084	0.0733 ± 0.0145
Stearic acid (C18:0)	11.78 ± 0.73	14.96 ± 0.59*
Elaidic acid (C18:1n9t) + Oleic acid (C18:1n9c)	15.74 ± 1.08	11.81 ± 1.04*
Arachidic acid (C20:0)	0.0379 ± 0.0035	0.0541 ± 0.0027*
*cis*-11-Eicosenoic acid (C20:1)	0.1586 ± 0.0166	0.1421 ± 0.0039
Heneicosanoic acid (C21:0)	0.2808 ± 0.0387	0.3486 ± 0.0225
*cis*-11,14-Eicosadienoic acid (C20:2)	0.0193 ± 0.0013	0.0197 ± 0.0012
Behenic acid (C22:0)	0.0147 ± 0.0013	0.0213 ± 0.0011*
Erucic acid (C22:1n9)	0.0118 ± 0.0031	0.0142 ± 0.0042
Tricosanoic acid (C23:0)	0.0099 ± 0.0028	0.0044 ± 0.0017
*cis*-13,16-Docosadienoic acid (C22:2)	0.0060 ± 0.0008	0.0089 ± 0.0007*
Lignoceric acid (C24:0)	0.0349 ± 0.0112	0.0259 ± 0.0016
Nervonic acid (C24:1)	0.1393 ± 0.0145	0.1582 ± 0.0073

The percent fatty acid compositions were calculated based on the total contents of 32 detected fatty acids. Data represent means ± SE (*n* = 6).

*p <0.05 vs. control group.

**Table 4:** Alterations in hepatic essential fatty acid composition in response to the HP diet.

	composition(%)	
Fatty acid	C	HP
Linoleic acid (C18:2n6c)	15.00 ± 1.17	17.43 ± 0.30
γ-Linolenic acid (C18:3n6)	0.1877 ± 0.0061	0.2192 ± 0.0108*
Linolenic acid (C18:3n3)	0.4989 ± 0.0522	0.5612 ± 0.0187
*cis*-8,11,14-Eicosatrienoic acid (C20:3n6)	0.5113 ± 0.0421	0.5610 ± 0.0145
*cis*-11,14,17-Eicosatrienoic acid (C20:3n3)	0.0312 ± 0.0042	0.0347 ± 0.0024
Arachidonic acid (C20:4n6)	16.98 ± 1.34	21.61 ± 1.01*
*cis*-5,8,11,14,17-Eicosapentaenoic acid (C20:5n3)	0.2690 ± 0.0262	0.3970 ± 0.0362*
*cis*-4,7,10,13,16,19-Docosahexaenoic acid (C22:6n3)	4.838 ± 0.471	5.547 ± 0.259

The percent fatty acid compositions were calculated based on the total contents of 32 detected fatty acids. Data represent means ± SE (*n* = 6).

*p <0.05 vs. control group.

### Upstream regulator analysis

**Fig 5:**
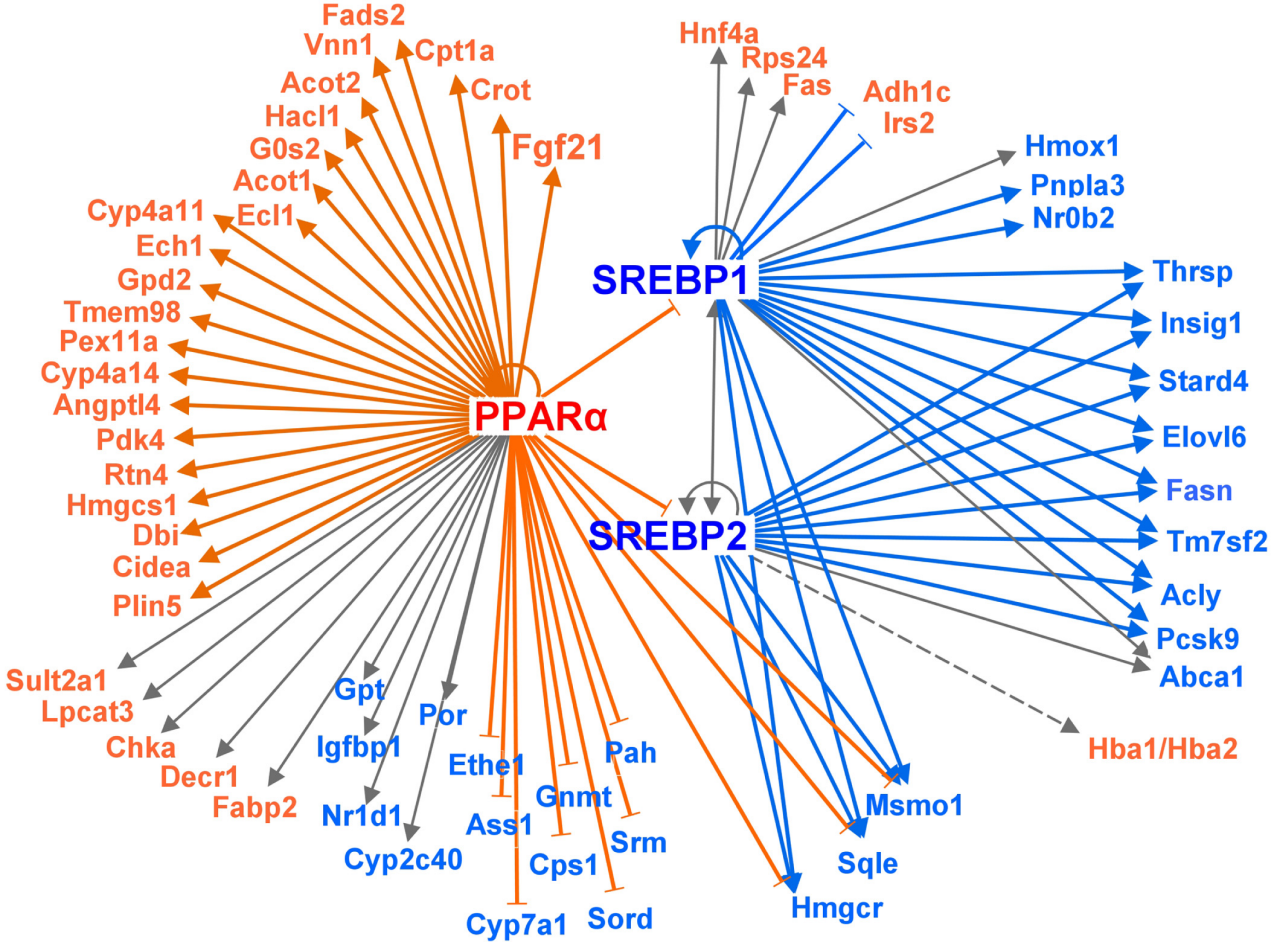
Evaluation of transcription factor activation involved in lipid metabolism. Enzymes and factors whose mRNA expression is higher and lower in the livers of high phosphorous (HP) diet-fed rats are shown in orange and blue, respectively, in comparison to their expression in the livers of control diet-fed rats. Gray lines indicate that it is unpredictable whether expression of the given enzyme/factor is increased or decreased by the HP diet. Dotted lines indicate indirect regulation. Activation of peroxisome proliferator-activated receptor alpha (PPARα) is predicted to be up-regulated (z-score >2), while activation of SREBP1 and SREBP2 is predicted to be down-regulated (z-score <2).

Fig 5 shows a bioinformatic evaluation of upstream regulators of DEGs using Ingenuity Pathway Analysis (IPA; Ingenuity H Systems, Redwood City, CA, USA; http://www.ingenuity.com). The IPA program consists of the Ingenuity Pathway Knowledge Base derived from known functions and published gene interactions. In this analysis, z-scores of >2 were regarded as significantly up-regulated, and those of <2 were regarded as significantly down-regulated. Activation of peroxisome proliferator-activated receptor alpha (PPARα) was predicted to be up-regulated (z-score >2) by DEGs, while activation of SREBP1 and SREBP2, which regulate the synthesis of fatty acids and cholesterol, respectively, was predicted to be down-regulated (z-score <2).

### FGF21 serum concentration

**Fig 6:**
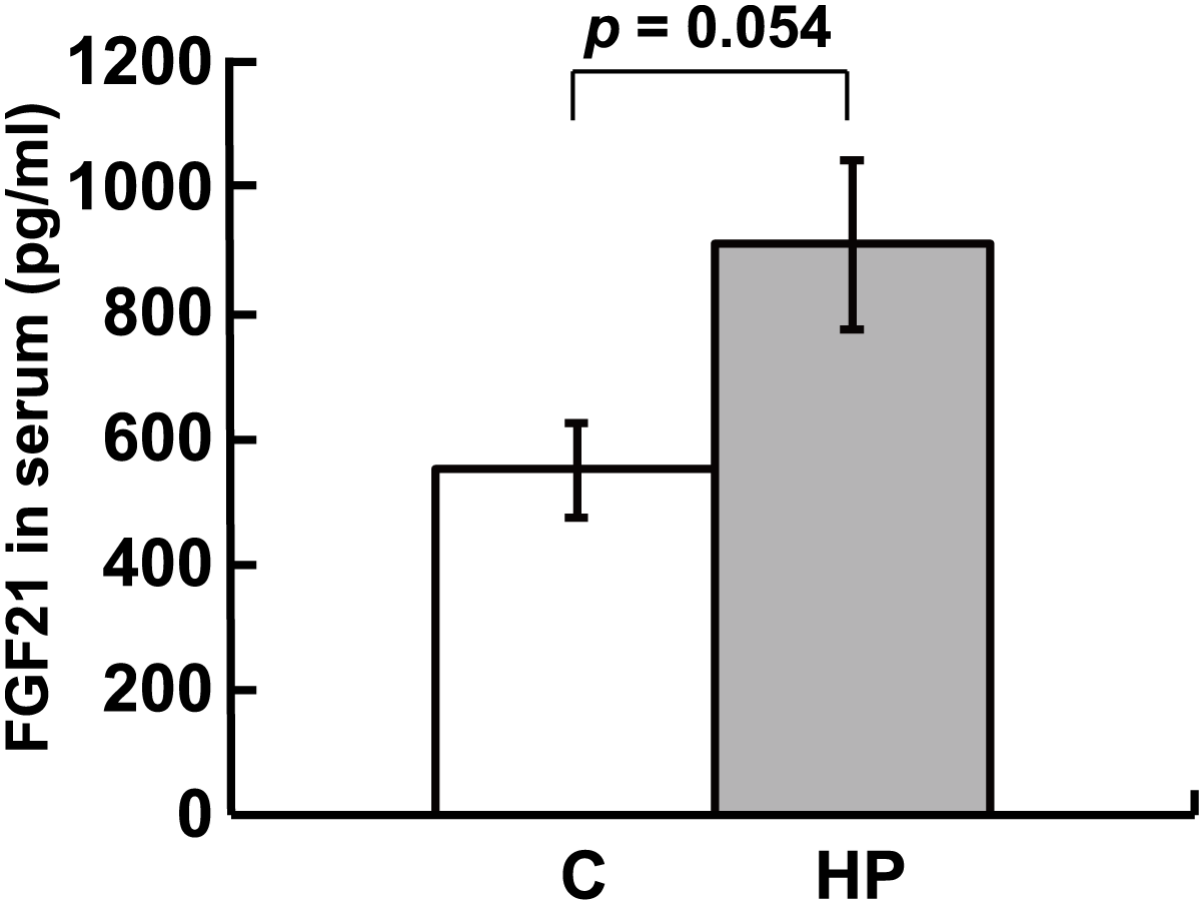
Concentration of serum fibroblast growth factor 21 (FGF21) in response to the high phosphorus (HP) diet. Data represent means ± standard error (*n* = 5 or 6). C, rats fed the control diet; HP, rats fed the HP diet.

In the DNA microarray data, *Fgf21* showed the fourth highest change in expression among up-regulated DEGs (S1 Table); therefore, we measured the FGF21 serum concentration by ELISA. The FGF21 serum concentration was higher in HP diet-fed rats (*p* = 0.054) than in control diet-fed rats (Fig 6).

### Change in white adipose tissue (WAT) weight

**Fig 7:**
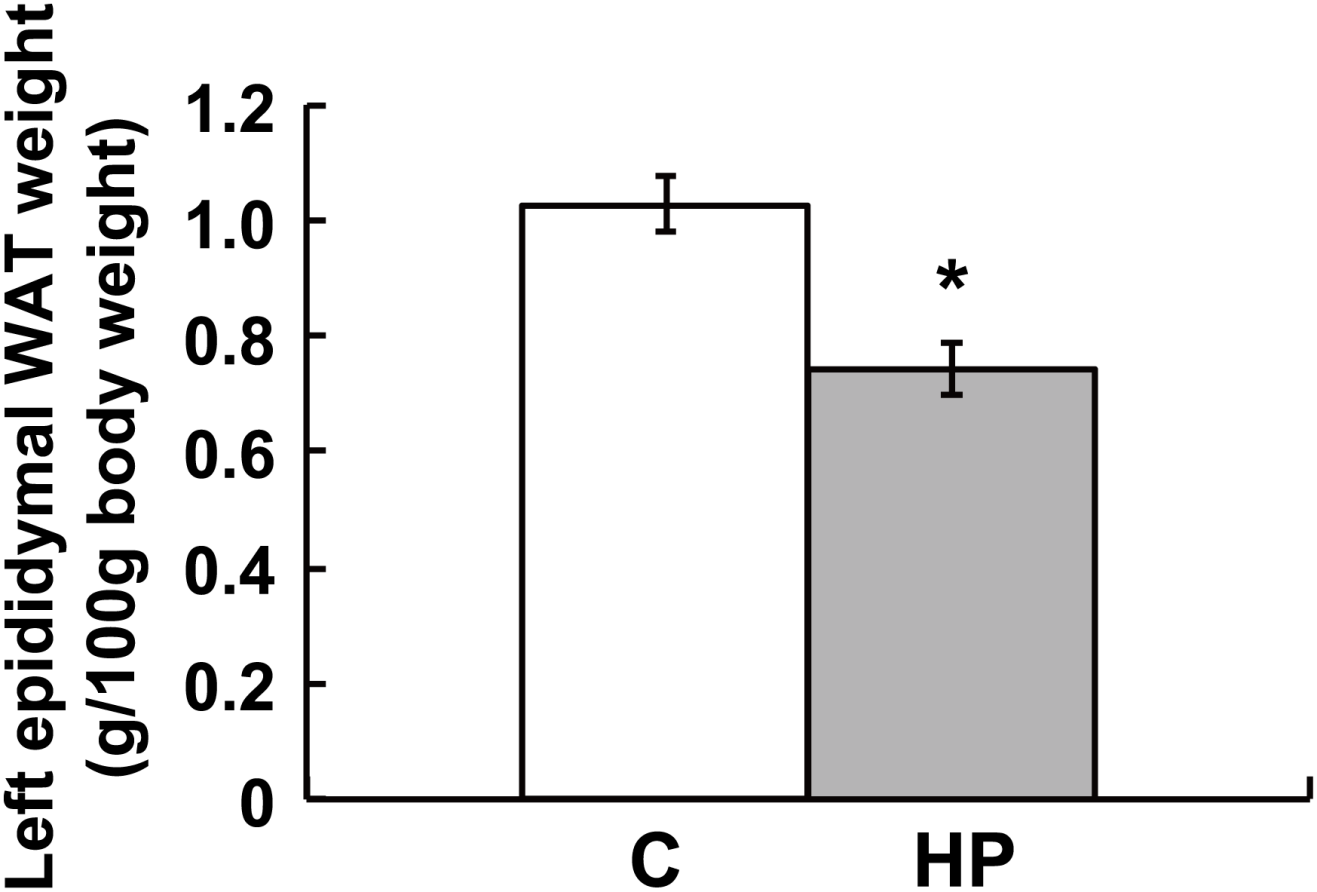
Change in weight of WAT in response to the high phosphorus (HP) diet. Data represent means ± standard error (*n* = 5 or 6). C, rats fed the control diet; HP, rats fed the HP diet.

We also measured the weight of epididymal WAT. The weight of WAT in rats fed the HP diet was significantly lower than in those fed the control diet (Fig 7).

## Discussion

In this study, we showed that a HP diet probably promotes energy expenditure via the utilization of fatty acids in rat liver; and we also confirmed that the weight of epididymal WAT was significantly lower in HP diet-fed rats. To our knowledge, there are no previous studies of the relationship between HP diet and energy metabolism, while many studies have investigated changes in homeostasis regulation in response to a HP diet. Our DNA microarray analysis revealed that fatty acid oxidation was enhanced in the livers of rats fed a HP diet. Moreover, the HP diet had a tendency to increase the serum concentration of FGF21, which promotes energy expenditure. These results suggest that a HP diet induces a fasting state-like hepatic gene expression profile.

Our previous study demonstrated that DNA microarray analysis is an appropriate method to comprehensively evaluate the effects of a HP diet on the kidney, such as the changes in mRNA expression levels of genes involved in calcification, fibrillization, and inflammation, reporting the novel effects of such a diet on phosphate homeostasis regulation [13]. In the present study, we used livers of animals that were the same as those used in this previous study. Rats were fed a diet containing 1.2% phosphorus, which is 4-fold higher than the amount of phosphorous in the AIN-93G control diet, for 24 days. There were no significant differences between rats fed the control diet and those fed the HP diet in terms of final food intake; accordingly, it was considered appropriate to evaluate the effects of the HP diet on hepatic gene expression using a DNA microarray. In our previous study, a large amount of phosphorus passed through the bodies of HP diet-fed rats, indicating that phosphorus absorption, as well as urinary phosphorus excretion, was significantly increased. Thus, it can be assumed that the livers of rats fed the HP diet are exposed to high concentrations of phosphates, at least transiently. We examined the expression data for known phosphate transporters, but found no significant change except for one probe set (Slc20a2; PiT-2) (S3 Table). Although rats have a bile duct for excretion, these data suggest that the liver does not contribute appreciably to phosphate homeostasis.

GO terms that were significantly enriched among DEGs belonged to two regarding categories: amino acid metabolism and lipid metabolism (Fig 1). The liver plays a pivotal role in energy homeostasis regulating carbohydrate, lipid, and amino acid metabolism, and there is cross-talk among these three metabolic pathways. In this study, we found that a HP diet altered the expression of genes involved in lipid and amino acid metabolic processes.

HP diets reduce nitrogen retention and protein synthesis [15, 19], which indicates that such a diet can affect amino acid metabolism. In the present study, DNA microarray analysis showed that genes encoding proteins that regulate amino acid catabolism and the urea cycle were down-regulated in the livers of rats fed the HP diet (S2 Table; Fig 2). The down-regulation of several aminotransferases, which catalyze amino group transfer in the catabolic pathway of Cys, Ser, Gly, Met, Trp, Phe, Tyr, and Val, indicates that amino acid catabolism was suppressed in rats fed the HP diet. Moreover, expression of *Cps1*, *Gpt1*, and *Gpt2* was down-regulated in the livers of HP diet-fed rats, showing that the urea cycle was suppressed. *Cps1* encodes an enzyme that catalyzes the committed step of the urea cycle, whereas *Gpt1* and *Gpt2* encode enzymes that catalyze the transamination reaction in the glucose-alanine cycle, which exports amino acid catabolites from muscle to the urea cycle [20]. These results suggest that a HP diet reduces the use of amino acids as an energy source.

Interestingly, the expression of a large number of genes involved in lipid metabolism was significantly changed in the livers of rats fed the HP diet (Table 1; Fig 3). Specifically, genes involved in fatty acid β-oxidation were up-regulated, whereas those involved in the biosynthesis of fatty acids and cholesterol were down-regulated. These changes in gene expression are similar to those that occur during fasting. The HP diet induced increased expression of *Cpt1a*, which encodes a rate-limiting enzyme for β-oxidation, together with that of many genes involved in the catabolism of fatty acids into acetyl-CoA. These results indicate that whole body energy was acquired via fatty acid β-oxidation in HP diet-fed rats. In addition, these data are in accordance with the down-regulation of genes related to fatty acid and cholesterol synthesis in HP diet-fed rats, including *Srebf1*, which encodes a transcription factor for the control of lipid biosynthesis, as well as *Fasn*, *Elovl6*, *Hmgcr*, *Sqle*, *Sc4mol*, and *Cyp7a1* (Fig 3). By contrast, the genes encoding Elovl2 and Fads2, which catalyze the elongation and desaturation reactions, respectively, of the essential fatty acids omega-3 and -6 were up-regulated in the livers of rats fed the HP diet. Taken together, these findings suggest that the fatty acid composition was altered in the livers of HP diet-fed rats. The proportion of essential fatty acids, including omega-3 and -6, was increased in the livers of HP diet-fed rats (Fig 4), which was owing to the increased concentrations of γ-linolenic acid, arachidonic acid, and EPA (Table 4). Omega-3 and -6 are agonists of PPARα, a transcription factor that regulates fatty acid β-oxidation. EPA activates PPARα robustly [21], and omega-3 and -6 suppress SREBP activity indirectly [22]. Taken together, our data suggest that a HP diet induces increased levels of omega-3 and -6 fatty acids and activation of PPARα, although it is unknown whether the former is a cause or a consequence of the latter. Moreover, IPA of upstream regulators revealed the up-regulation of *PPARα* and the down-regulation of *SREBP1* and *SREBP2*, which encode transcription factors involved in the regulation of fatty acid and cholesterol synthesis, respectively, in the livers of HP diet-fed rats based on changes in the expression of their target genes (Fig 5). This bioinformatic evaluation indicates that alteration of the gene expression profile, such that fatty acid metabolism is increased, is regulated by the aforementioned upstream transcription factors in response to the HP diet. It was difficult to determine whether the HP diet directly altered hepatic fatty acid composition in the current study; however, the increased levels of omega-3 and -6 fatty acids indicate that fatty acid metabolism was increased. Further studies are required to explain the correlation between increased levels of omega-3 and -6 fatty acids and fatty acid β-oxidation induced by a HP diet.

There were no statistically significant changes in the serum concentrations of NEFA and T-KB, which are byproducts in the breakdown of fatty acids to generate energy in the liver, between rats fed the HP diet and those fed the control diet (S2 Fig). In addition, we examined whether hepatic expression of Cpt1a and Fasn protein was altered by the HP diet. However, there were no statistically significant changes in the expression of these genes at the protein level (S4 Fig). We postulate that the effects of a diet containing 1.2% phosphorous on lipid metabolism accumulate gradually over a period of 24 days; therefore, we could not detect the changes in serum concentrations of lipid markers at 24 days.

Next, we focused on a PPARα target gene, *Fgf21*, because it could be an important indicator of energy expenditure. The hormone FGF21 is secreted from the liver into the blood and increases energy expenditure [23]. FGF21 is an atypical member of a subfamily of FGFs that function as endocrine hormones [24, 25], is involved in the regulation of adipocyte lipolysis, and inhibits hepatic lipogenesis [26, 27]. In our DNA microarray analysis, *Fgf21* showed the fourth highest change in expression among up-regulated DEGs (S1 Table); therefore, we measured the FGF21 serum concentration by ELISA. The FGF21 serum concentration was higher (*p* = 0.054) in rats fed the HP diet than in those fed the control diet (Fig 6). This suggests that the HP diet enhanced the utilization of free fatty acids released by lipolysis of WAT. Indeed, the weight of WAT was significantly decreased in rats fed the HP diet (Fig 7). On the other hand, serum glycerol concentration did not change significantly (S3 Fig) and this observation coincided with those of serum TG and NEFA. These results suggest that the lipolysis of WAT is a gradual change, while glycerol and NEFA produced by HP diet-induced lipolysis are rapidly catabolized.

*In vivo* FGF21 expression was recently shown to be induced by environmental and tissue stresses such as fasting [28], cold exposure, autophagy deficiency in the liver [29], and PPARα agonist treatment [30]. HP diets induce mineral imbalance [31–33] and nitrogen imbalance [15, 19], and could therefore be regarded as an environmental stress. Therefore, a HP diet likely enhances energy expenditure through fatty acid utilization by inducing FGF21 expression.

In summary, our DNA microarray analysis revealed that a HP diet promotes energy expenditure through fatty acid utilization in rat liver. In accordance with this, we confirmed that a HP diet increased the serum concentration of FGF21. Moreover, we observed changes in fatty acid composition that corresponded to changes in the transcriptome, namely, increased hepatic levels of omega-3 and -6 fatty acids. Although further studies are required to clarify the molecular mechanism linking the dietary phosphate level to fatty acid metabolism, these findings provide novel insights into the physiological effects of a HP diet.

## Materials and Methods

### Animals and diets

Liver and blood samples acquired in our previous study [13] were used in the current study. In brief, 4-week-old male Wistar rats were individually housed in metabolic cages under controlled conditions of 22 ± 1°C and a 12 hour light/dark cycle (lights on from 08:00 to 20:00 daily). The control diet, which contained 0.3% phosphorous, and the HP diet, which contained 1.2% phosphorous, were prepared based on the composition of the AIN-93G diet [34]. After 7 days of acclimatization, rats were divided into two groups (n = 5 per group) and fed the control or HP diet for 24 days. The animals were allowed access to food *ad libitum* and had free access to water (Milli-Q). On Day 24, all rats were sacrificed under anesthesia, and blood and liver samples were taken for analysis. Serum samples were stored at −20°C, and tissue samples were frozen in liquid nitrogen immediately after excision and stored at −80°C until use. For measurements of fatty acid composition, serum FGF21 and glycerol concentrations, and epididymal WAT weight, an additional experiment was performed (n = 6 or 7) as described above. Serum biochemical parameters were similar in both experiments; therefore, the data presented are from the latter. The protocol for animal experiments was approved by the Animal Use Committee of the Faculty of Agriculture at The University of Tokyo (approval numbers: P09-283 and P11-597).

### DNA microarray

Total RNA was isolated from liver using TRIzol reagent (Invitrogen Life Technologies, Carlsbad, CA) and purified using an RNeasy Mini Kit (Qiagen K.K., Tokyo, Japan). The quality and quantity of purified total RNA were confirmed by agarose gel electrophoresis and spectrophotometry, respectively. DNA microarray analysis of the liver was performed for four rats that had undergone DNA microarray analysis of the kidney in our previous study [13]. DNA microarray analysis was performed as described previously [13]. All microarray data are MIAME compliant and have been deposited in a MIAME compliant database, the National Center for Biotechnology Information (NCBI) Gene Expression Omnibus (http://www.ncbi.nlm.nih.gov/geo/, GEO Series accession number GSE71201), as detailed on the FGED Society website (http://fged.org/projects/miame/).

### DNA microarray data analysis

CEL files were quantified with FARMS [17] using statistical language R [35] (http://www.r-project). Principal component analysis [36] was performed using the prcomp() function in R. The RP method [18] was applied to quantified data to identify DEGs, with the number of permutations set at 1,000. Probe sets presenting an FDR <0.05 were regarded as having significantly different expression levels between the two groups.

Gene-annotation enrichment analysis of DEGs was performed using the Database for Annotation, Visualization, and Integrated Discovery (DAVID; http://david.abcc.ncifcrf.gov/) [37] and QuickGO (http://www.ebi.ac.uk/QuickGO/) [38]. EASE scores, which are modified Fisher’s exact test *p*-values [39], were used to evaluate statistically over-represented GO terms from the DEGs. Benjamini and Hochberg FDR corrections for multiple testing [40] were used to correct the results. GO terms with FDR-corrected *p-*values of <0.05 were regarded as significantly enriched.

To evaluate alterations in transcription factor activation, DEGs were imported into the IPA tool (Ingenuity H Systems), and upstream regulator analysis was performed. A z-score of >2 was regarded as significantly up-regulated, and a z-score of <2 was regarded as significantly down-regulated.

### Fatty acid composition analysis

Freeze-dried rat liver samples were homogenized using a ball mill mixer (MM301; Retsch, Haan, Germany) for 3 min at 20 Hz with cooling using liquid nitrogen. Each 5 mg of homogenate was spiked with 100 μL of internal standard solution (1 mg/mL tridecanoic acid prepared in a 1:1 (v/v) solution of chloroform:methanol). A fatty acid methylation kit (Nacalai Tesque, Japan) was used to methylate and purify fatty acids in liver samples. The resultant solution was subjected to gas chromatography (GC)/mass spectrometry (MS) to measure fatty acid methyl esters.

GC/MS analyses were performed using an Agilent 7000B GC/MS TripleQuad system (Agilent Co., Palo Alto, CA, USAh). In the GC system, an OMEGAWAXTM capillary column (30 m × 0.25 mm I.D., film thickness: 0.25 μm; Sigma-Aldrich, St. Louis, MO) was used. The GC column temperature was held at 50°C for 2 min and then increased to 230°C at a rate of 4°C/min and maintained for 15 min. The inlet temperature was maintained at 250°C. The flow rate of helium gas through the column was 1 ml/min. Aliquots of 1.0 μl were injected with the split. The MS conditions were as follows: ionization voltage: 70 eV; ion source temperature: 250°C; and full scan mode in the *m*/*z* of 50–650. The identities of each peak in the chromatogram were confirmed by comparison with the mass spectra and retention times of authentic standards (FAME Mix 75m SP-2560, Sigma-Aldrich).

### Measurement of lipids and FGF21 in serum

T-CHO, E-CHO, TG, PL, LDL-C, HDL-C, NEFA, and T-KB were measured by Nagahama Life Science Laboratory (Shiga, Japan). FGF21 was measured using an ELISA kit (Biovendor, Czech Republic).

### Statistical analysis

Between-group differences except for those in the DNA microarray data were considered significant at *p* < 0.05, using a non-paired Student’s *t*-test.

## Supporting Information

S1 Fig Principal component analysis of FARMS-quantified DNA microarray data. HP, high phosphorus diet group; C, control diet group. Numbers represent independent samples. The labels on the x- and y- axes represent PC1 and PC2, respectively with proportion of variance.
(PDF)

S2 Fig Serum lipid concentrations in response to a HP diet. (PDF)

S3 Fig Serum glycerol concentrations in response to a HP diet. Serum glycerol concentrations were measured using Glycerol Colorimetric Assay kit (Cayman, Ann Arbor, MI, USA). C, rats fed the control diet; HP, rats fed the HP diet.
(PDF)

S4 Fig Western blot analysis of Cpt1a and FAS protein. Hepatic expression of Cpt1a (A) and FAS (B) were analyzed by Western blotting. Left, the entire membrane image. Right, quantified band intensities. Data represent means ± standard error (*n* = 5). C, rats fed the control diet; HP, rats fed the HP diet. Details of methods are described in S1 Materials and Methods.
(PDF)

S1 Materials and Methods Supplemental description for Western blot analysis. (DOCX)

S1 Table A full list of differentially expressed probe sets. (XLSX)

S2 Table Genes related to amino acid metabolism whose expression was altered in response to a HP diet. (PDF)

S3 Table Gene expression data of known major phosphate transporters. (XLSX)
