# Supplementary figures and images for: A High Phosphorus Diet Affects Lipid Metabolism in Rat Liver: A DNA Microarray Analysis
Source: PLoS One. 2016 May 17;11(5):e0155386. doi: 10.1371/journal.pone.0155386 (PMC4871335; doi:10.1371/journal.pone.0155386)

# PCA

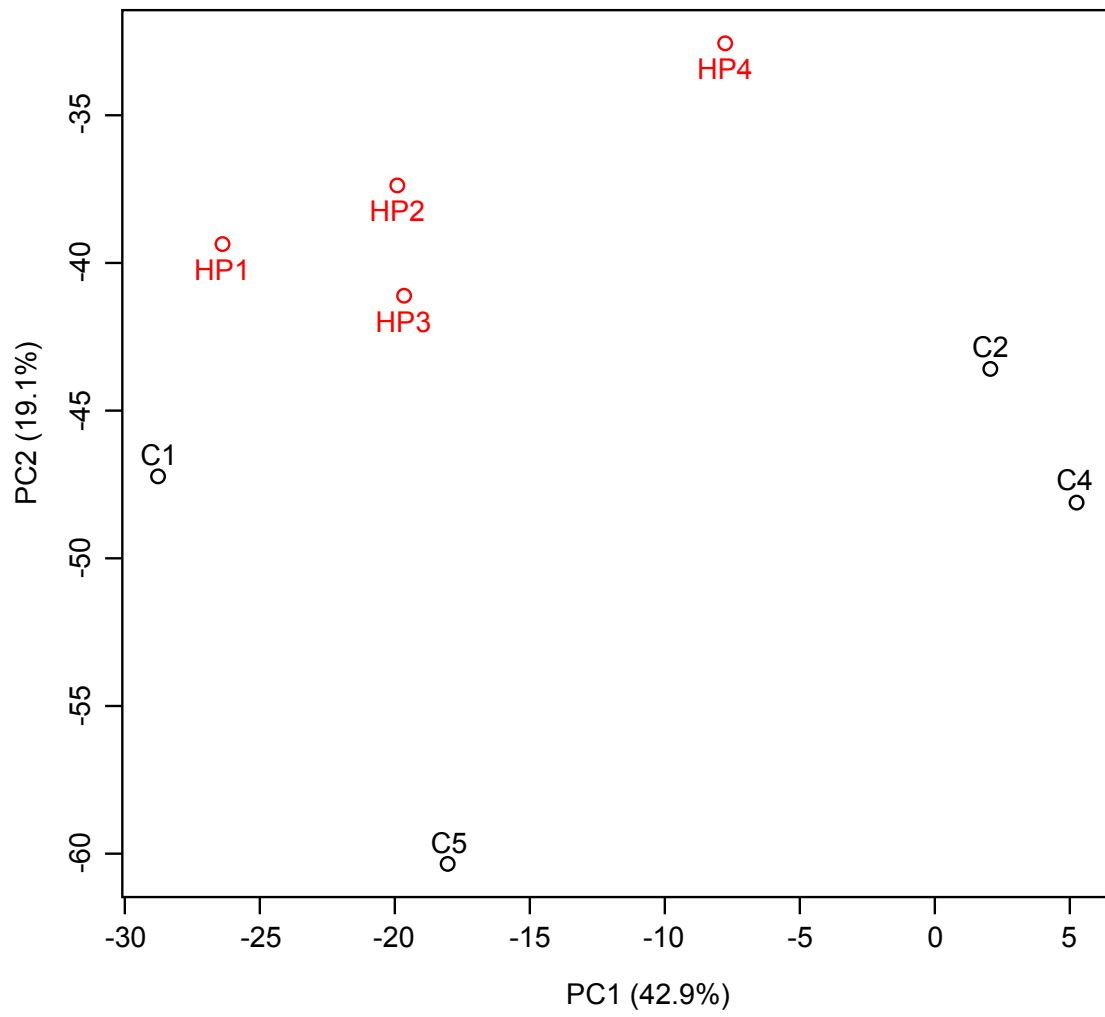

Supplement: S1 Fig — HP, high phosphorus diet group; C, control diet group. Numbers represent independent samples. The labels on the x- and y- axes represent PC1 and PC2, respectively with proportion of variance. (PDF) [file pone.0155386.s001.pdf]

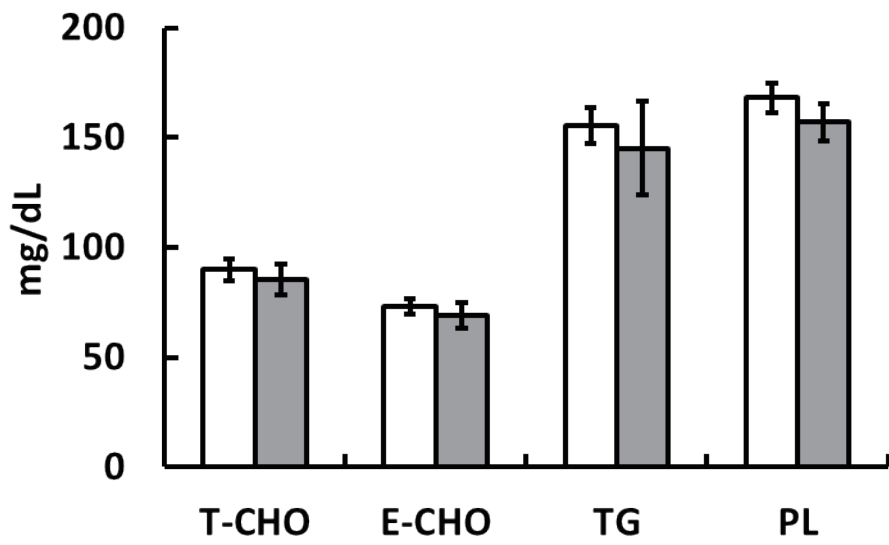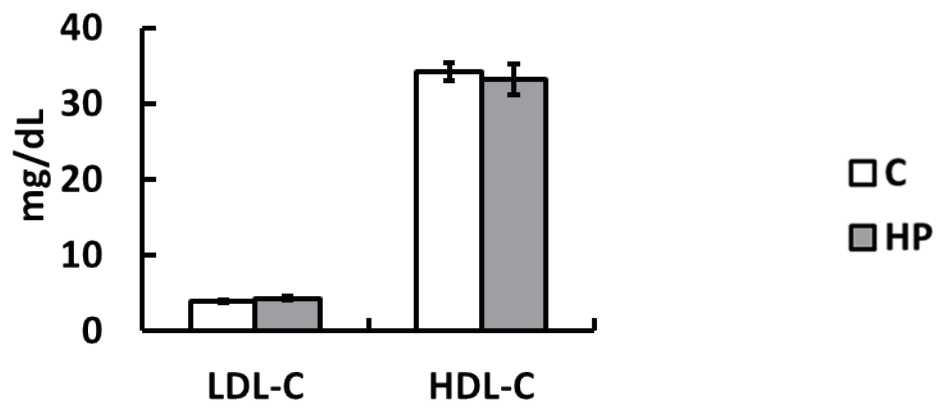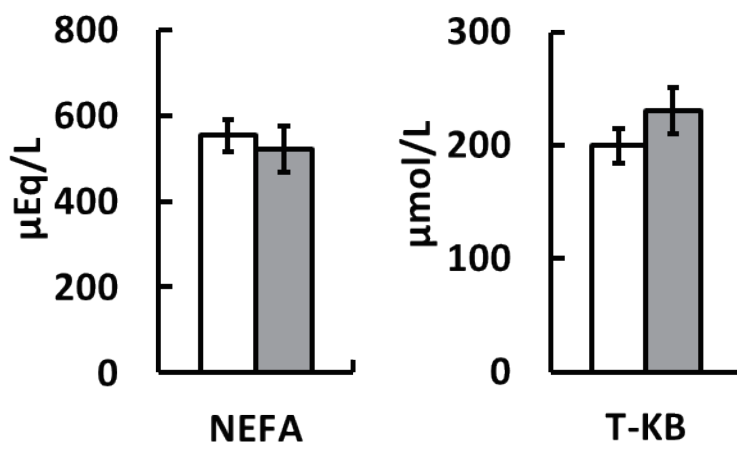

Supplement: S2 Fig — (PDF) [file pone.0155386.s002.pdf]

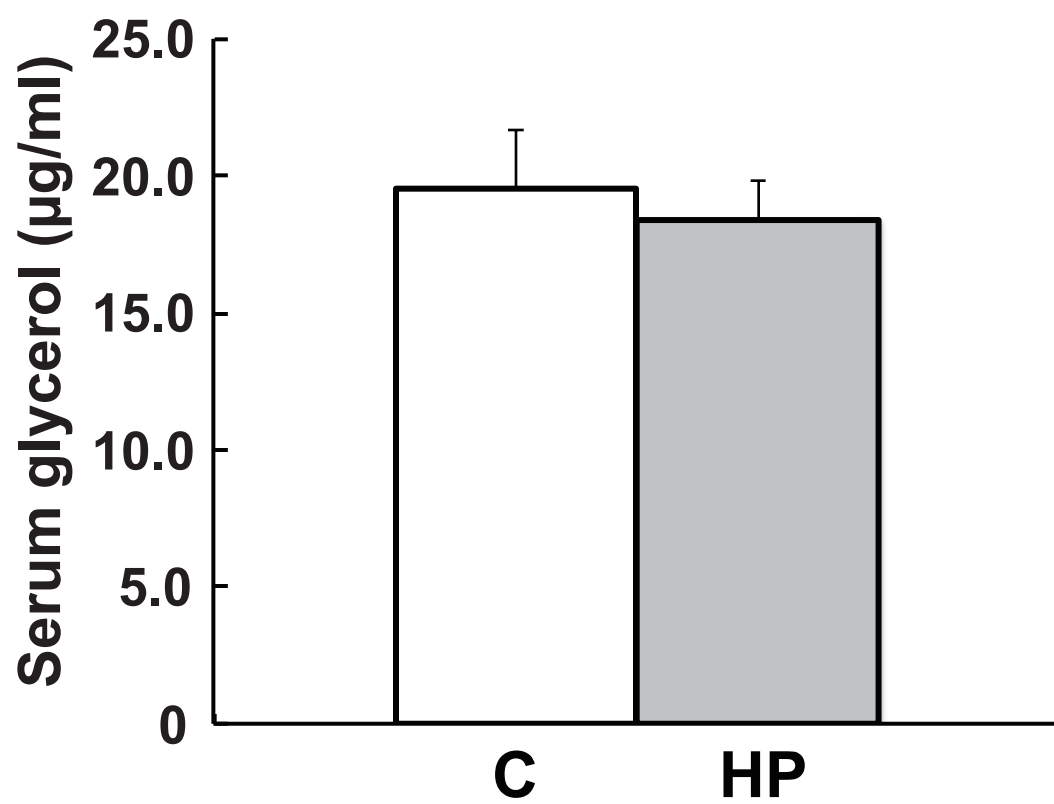

Supplement: S3 Fig — Serum glycerol concentrations were measured using Glycerol Colorimetric Assay kit (Cayman, Ann Arbor, MI, USA). C, rats fed the control diet; HP, rats fed the HP diet. (PDF) [file pone.0155386.s003.pdf]

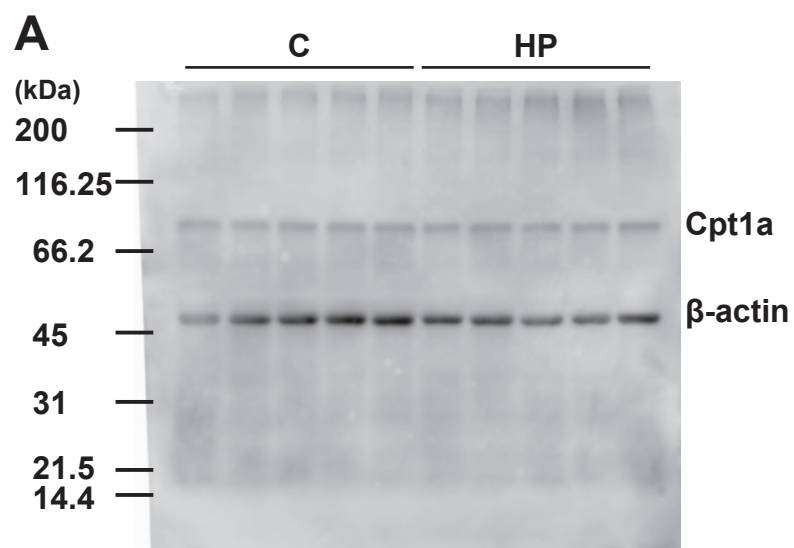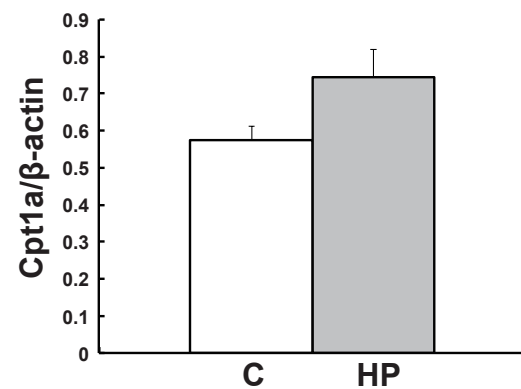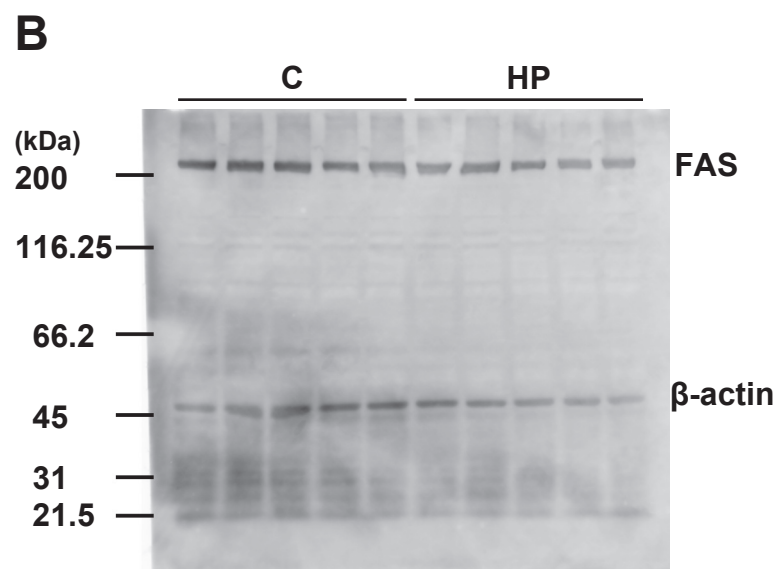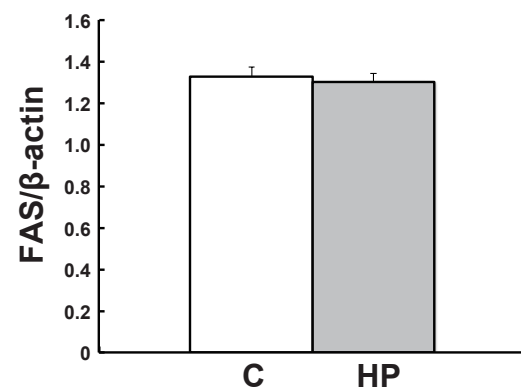

Supplement: S4 Fig — Hepatic expression of Cpt1a (A) and FAS (B) were analyzed by Western blotting. Left, the entire membrane image. Right, quantified band intensities. Data represent means ± standard error (n = 5). C, rats fed the control diet; HP, rats fed the HP diet. Details of methods are described in S1 Materials and Methods. (PDF) [file pone.0155386.s004.pdf]
