# Supplementary material for: A High Phosphorus Diet Affects Lipid Metabolism in Rat Liver: A DNA Microarray Analysis
Source: PLoS One. 2016 May 17;11(5):e0155386. doi: 10.1371/journal.pone.0155386 (PMC4871335; doi:10.1371/journal.pone.0155386)
Supplement: S1 Materials and Methods — (DOCX) [file pone.0155386.s005.docx]

**S1 Materials and Methods**

**Materials**

Anti-carnitine palmitoyltransferase 1a (Cpt1a), anti-fatty acid synthase (FAS), anti-β-actin, horseradish peroxidase (HRP)-conjugated anti-mouse IgG, and HRP-conjugated anti-rabbit IgG antibodies were purchased from Abcam (Cambridge, UK). Other chemicals were purchased from Nacalai tesque (Kyoto, Japan).

**Western blotting**

Frozen rat liver sample (approx. 100 mg) was homogenized with Polytron homogenizer (Kinematica, Luzern, Switzerland) in 1 ml of lysis buffer [30 mM Tris-HCl, pH7.6, 150 mM NaCl, 1% Triton X-100, 10% glycerol] containing 1/100 vol. of protease inhibitor cocktail. The lysate was centrifuged at 13,200 × g for 10 min at 4°C, and then the resultant supernatant was subjected to SDS-PAGE. Proteins were transferred to an Immun-Blot^®^ PVDF membrane (Bio-Rad, Hercules, CA, USA), and the membrane was blocked with 5% BSA (Cohn Fraction V, Sigma-Aldrich, St. Louis, MO, USA) in Tris-buffered saline for 1 h. The membrane was then incubated with anti-β-actin and anti-Cpt1a, or anti-FAS antibody and washed prior to incubation with HRP-conjugated secondary antibody. Signals were visualized by chemiluminescence reactions with the SuperSignal™ West Dura kit (Thermo Scientific, Rockford, IL, USA), and the images were processed using an ImageQuant LAS 4000 lumino-image analyzer (GE Healthcare, Buckinghamshire, UK). The images were analyzed with ImageQuant TL software (GE Healthcare) to determine band intensities.
