# Supplementary material for: A High Phosphorus Diet Affects Lipid Metabolism in Rat Liver: A DNA Microarray Analysis
Source: PLoS One. 2016 May 17;11(5):e0155386. doi: 10.1371/journal.pone.0155386 (PMC4871335; doi:10.1371/journal.pone.0155386)
Supplement: S2 Table — (PDF) [file pone.0155386.s007.pdf]

S2 Table. Genes related to amino acid metabolism whose expression was altered in response to a HP diet.

Probe ID	Gene Symbol	Gene Title	Average Difference	Expression
**Amino acid catabolism**				
1397924_at	*Hibch*	3-hydroxyisobutyryl-Coenzyme A hydrolase	1.77E-01	up
1372340_at	*Mat2b*	methionine adenosyltransferase II, beta	1.39E-01	up
1369864_a_at	*Sds*	serine dehydratase	-1.11E+00	down
1387178_a_at	*Cbs*	cystathionine beta synthase	-1.76E-01	down
1370936_at	*Dmgdh*	dimethylglycine dehydrogenase	-2.24E-01	down
1368272_at	*Got1*	glutamic-oxaloacetic transaminase 1, soluble (aspartate aminotransferase 1)	-5.17E-01	down
1376427_a_at	*Gldc*	glycine dehydrogenase (decarboxylating)	-2.04E-01	down
1370811_at	*Mpst**	mercaptopyruvate sulfurtransferase	-2.29E-01	down
1387665_at	*Bhmt*	betaine-homocysteine S-methyltransferase	-4.02E-01	down
1387672_at	*Gnmt**	glycine N-methyltransferase	-1.76E-01	down
1368720_at	*Tdo2*	tryptophan 2,3-dioxygenase	-1.94E-01	down
1396300_at, 1396301_x_at,1394479_at	*Afmid**	arylformamidase	-2.58E-01	down
1387034_at	*Pah*	phenylalanine hydroxylase	-2.36E-01	down
1369790_at	*Tat*	tyrosine aminotransferase	-8.14E-01	down
1398514_at	*Hgd*	homogentisate 1, 2-dioxygenase	-2.27E-01	down
1387307_at	*Hal*	histidine ammonia lyase	-2.14E-01	down
1368814_at	*Aldh6a1*	aldehyde dehydrogenase 6 family, member A1	-2.12E-01	down
1372920_at	*Prodh*	proline dehydrogenase (oxidase) 1	-3.96E-01	down
**Urea cycle**				
1370151_at	*Cps1**	carbamoyl-phosphate synthetase 1	-2.65E-01	down
1370964_at	*Ass1*	argininosuccinate synthase 1	-2.41E-01	down
1368272_at	*Got1*	glutamic-oxaloacetic transaminase 1, soluble (aspartate aminotransferase 1)	-5.17E-01	down
1370375_at	*Gls2**	glutaminase 2 (liver, mitochondrial)	-3.12E-01	down
1387052_at	*Gpt**	glutamic-pyruvate transaminase (alanine aminotransferase)	-2.13E-01	down
1384903_at	*Gpt2**	glutamic pyruvate transaminase (alanine aminotransferase) 2	-2.03E-01	down
1367729_at	*Oat**	ornithine aminotransferase	-1.95E-01	down
1372920_at	*Prodh*	proline dehydrogenase (oxidase) 1	-3.96E-01	down
**Others**				
1387725_at	*Gulo*	gulonolactone (L-) oxidase	-2.98E-01	down

All genes were included in GO terms that were significantly enriched ($p<0.05$) in DEGs (Figure 1), except *DEG not included in selected GO terms but showed the identical function.
